# Transgender outpatient clinics within the SUS: how can communication enhance service visibility?

**DOI:** 10.1590/S2237-96222024v33e2024270.especial.en

**Published:** 2024-01-10

**Authors:** Juliana Michelotti Fleck, Salete Saionara Santos Barbosa, Aline Guio Cavaca

**Affiliations:** 1 Ministério da Saúde, Secretaria de Atenção Primária à Saúde, Departamento de Estratégias e Políticas de Saúde Comunitária, Brasília, DF, Brasil; 2 Ministério da Saúde, Secretaria de Vigilância em Saúde e Ambiente, Departamento de HIV/aids, tuberculose e hepatites virais e infecções sexualmente transmissíveis. Brasília, DF, Brasil; 3 Fundação Oswaldo Cruz, Escola de Governo Fiocruz-Brasília. Brasília, DF, Brasil

**Keywords:** Comunicación en Salud;, Transexualidad, Vulnerabilidad en Salud, Sistema Único de Salud, Personas Transgénero, Health Communication, Transsexuality, Health Vulnerability, Brazilian National Health System, Transgender People

## Abstract

**Objective:**

To map communication barriers within healthcare services for transgender people, and propose strategies to enhance the visibility of these outpatient clinics and improve access.

**Methods:**

This was a mixed-methods study, employing both quantitative and qualitative approaches. 44 online questionnaires were administered and two focus groups were conducted with transgender people and healthcare professionals in 2021.

**Results:**

the primary barriers identified regarding access to healthcare services included a lack of knowledge about the free and specialized nature of services, alongside the stigma and discrimination faced by the transgender population. Based on this diagnosis, a communication plan was developed, tailored to the needs of each population. The strategy was launched on National Trans Day of Visibility and emphasized the message “TRANS Path. Respect, Dignity and True Identity”.

**Conclusion:**

Developing communication strategies through a dialogical approach can increase the visibility of transgender clinics. This strategy includes the integration of users and professionals in collaborative practices, respecting gender identity and promoting more equitable access to services.

## Study contributions

**Main results:**

A dialogic communication strategy was developed in collaboration with both healthcare professionals and service users at trans outpatient clinics. This strategy was widely disseminated on social media during Brazil’s National Trans Day of Visibility, giving visibility and a voice to trans individuals.

**Implications for services:**

Health services should increase awareness of the free and specialized care provided by transgender outpatient clinics, train professionals to combat stigma, facilitate user access and adopt a dialogic communication approach that respects gender identity.

**Perspectives:**

The expansion of collaborative initiatives with the transgender population that promote inclusive policies can enhance visibility and equitable access to healthcare services.

## RESUMEN

**Objetivo:**

Mapear las dificultades de comunicación en los servicios de salud para personas transexuales, proponiendo estrategias para aumentar la visibilidad de estos centros y mejorar el acceso a ellos.

**Métodos:**

Se trata de un estudio mixto, con enfoques cuantitativos y cualitativos. En 2021, se aplicaron 44 cuestionarios en línea y se realizaron dos grupos focales con personas trans y profesionales de la salud.

**Resultados:**

La falta de conocimiento sobre la gratuidad y especialización de los servicios, así como el estigma y la discriminación que enfrenta la población trans, fueron las principales dificultades identificadas en relación con el acceso a los servicios de salud. A partir de este diagnóstico, se elaboró un plan de comunicación adaptado a las necesidades de cada grupo. La estrategia se lanzó en el Día Nacional de la Visibilidad Trans y enfatizó el mensaje: “Camino TRANS. Respeto, Dignidad y Identidad Verdadera”.

**Conclusión:**

El desarrollo de estrategias de comunicación de manera dialógica puede aumentar la visibilidad de los servicios de los centros para personas trans. Parte de esta estrategia es la integración de usuarios y profesionales en prácticas colaborativas, respetando la identidad de género y promoviendo un acceso más equitativo a los servicios.

## INTRODUCTION

The term “trans population” encompasses people whose gender identity differs from the sex assigned at birth. It refers to a person’s internal and individual experience of gender. This experience includes personal perceptions of the body, which may involve physical modifications through medical, surgical, or other means, as well as gender expressions such as clothing and mannerisms.^1^

Transsexual and transgender people are part of a diverse and vulnerable group. They face significant challenges, including a life expectancy of 35 years on average. Contributing to this concerning scenario are mental health issues, an increased risk of contracting HIV and other sexually transmitted infections, and social stressors such as violence, employment difficulties, and barriers to healthcare, all of which raise the likelihood of suicidal behavior.^2^

Due to healthcare professionals’ lack of knowledge about transsexuality, trans people often face negative experiences within healthcare services,^3^ exacerbated by difficult communication and acceptance barriers. The importance of offering specialized services that understand the need for body changes and the transsexualizing pathway as both health and disease processes is evident. In addition, the qualification of professionals to meet the needs of these individuals within the public health services is crucial.^4^

In 2013, a care pathway was established to provide healthcare to individuals undergoing gender transition within the Brazilian National Health System (*Sistema Único de Saúde* - SUS)^5^ This health care structure includes both primary care and specialized care. Primary care coordinates continuous care, while specialized care is responsible for urgent care services, outpatient, and hospital services. This involves clinical follow-up, pre- and post-operative care, hormone therapy, and surgeries. The transgender outpatient clinics within the SUS offer essential services and support spaces for the population undergoing transition or seeking information for self-acceptance. The support includes psychologists, speech therapists, nutritionists, endocrinologists, psychiatrists, and social workers, along with monitoring for hormone therapy.^5^

Despite the significance of these services, challenges remain in achieving excellence. Communication difficulties between health professionals and users at transgender outpatient clinics stand out, such as linguistic differences, physical limitations, imposition of values and unconscious influence.^6^ Sociocultural differences, stigmas and prejudices further complicate the process. This study aimed to identify these challenges and propose communication strategies to promote the visibility of transgender outpatient clinics and facilitate access to the services they provide.

## METHODS

**Figure 1:**
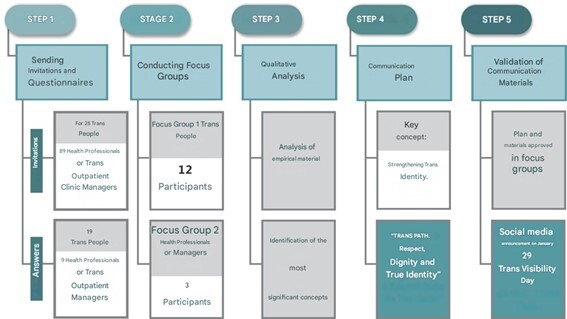
Methodological summary of the study.

This research employs a mixed-methods approach of both quantitative and qualitative nature, characterized as exploratory. It was carried out in five stages: sending targeted invitations for participation and application of questionnaires, conducting focus groups, reporting on the most significant points and qualitative analysis of the conducted groups, planning and proposing communication material and validating the materials produced (Figure 1).

### Setting and study population

The study involved two groups: trans people seeking health care in the SUS and health professionals working in transgender outpatient clinics and referral centers. Participants were selected based on information collected from the websites of the Ministry of Health, transgender outpatient clinics, hospitals, and LGBTQIAP+ nongovernmental organizations. Initial contacts were directed toward individuals from the five regions of the country. Potential subjects were invited through referrals from the initial participants, using the “snowball sampling technique’.^7^

The inclusion criteria for trans people included: being a trans man or woman, either a user or non-user of health services, selected through referral from outpatient clinics or organized networks of trans people. The inclusion criterion for health professionals was being employed at transgender outpatient clinic within the SUS, as a doctor, nurse, nursing assistant, psychologist or social worker. Transgender people under 18 years of age and health professionals serving solely as volunteers in transgender outpatient clinics were excluded from the study.

Due to the socio-health context of the COVID-19 pandemic, initial contact with participants and meetings were conducted via email, confirmed through the WhatsApp messaging application and by telephone. The focus groups took place online on the Zoom platform. After the invitation and confirmation of acceptance, participants were instructed to complete an online questionnaire, sent 15 days prior the focus groups sessions, and to sign the Free and Informed Consent Form (FICF).

### Data collection

Questionnaires were distributed via Google Forms through email and WhatsApp to gather information regarding the profile of interested parties and their perspectives on the research topic.

A total of 9 responses were received from healthcare professionals and 19 from transgender people after 89 and 25 invitations were sent. The questionnaires, which included both open-ended and multiple-choice questions, were tailored for each group. They addressed aspects such as identification, location, age, education, field of work, use and performance in outpatient services, and type of communication used in transgender outpatient clinic services. 

All invited respondents were divided into two focus groups: one comprising transgender people (focus group 1) and another consisting of health professionals (focus group 2). Focus group 1 included 12 people from four regions of the country (Midwest, Southeast, North and Northeast), in addition to two moderators and a technical support staff member. Focus group 2 had three health professionals from the Midwest, Southeast and Northeast regions, in addition to two moderators and a professional for support and technical assistance. The focus groups were conducted virtually, lasting approximately two hours and thirty minutes. The study followed the guidelines of Circular Letter No. 2/2021 of the National Research Ethics Committee^9^ concerning research conducted in virtual environments. The research was approved by the Research Ethics Committee of Fiocruz Brasília (CAAES 50379321.1.0000.802**7**)**,** ensuring ethics, confidentiality and clarity regarding the procedures.

### Data analysis

The questionnaire data were analyzed descriptively to characterize the study population, and the transcripts from focus group were subjected to thematic content analysis to interpret the communications.^8^ Participants’ impressions and experiences regarding life stories, outpatient clinic settings, and communication were compared. Common themes included participant’s history, knowledge of transgender outpatient clinic services, and communication.

### Development of the communication plan

The focus groups identified key points in communication, information gaps, and effective strategies to enhance the visibility of transgender outpatient clinics. Based on these results, a communication plan was developed that included materials and engagement strategies for institutions, users, and professionals. A national mobilization via social media was suggested, led by the focus group participants, with messages shared on the Instagram profile @comunicatrans.

### Validation of materials produced

The validation of the communication plan took place in a two-hour virtual meeting, via Microsoft Teams, with a group of 12 people that included users and health professionals from the focus groups. The communication strategy and the campaign concept were presented, and there were suggestions for mobilization and shareable materials. The materials were disseminated, evaluated, and adjustments proposed by participants, accompanied by discussions regarding the applicability of the produced materials.

## RESULTS

## Brief description of participants

According to the analysis of the 19 questionnaires from the transgender population, 5 respondents were trans men and 14 were trans women, the majority of whom were young (11 individuals aged 18 to 39 years). All participants were already users of the SUS, 14 of whom were users of the trans outpatient clinics. Three respondents were unaware of the services provided by these outpatient clinics. The average level of education of this group was complete higher education. Two people had only completed high school.

According to the questionnaires completed by the 9 healthcare professionals, all were directly linked to transgender outpatient clinics. Five were cisgender women, and 4 were cisgender men. Four respondents were aged 30 to 39 years, and 4 were 50 years old or older. Two respondents were doctors, and the others reported working in related fields.

## FOCUS GROUP 1 – TRANSGENDER PEOPLE

### Category 1: “portrait”

The activity began with the “portrait” category. In this category, each participant introduced themselves and shared their life stories. Personal experiences regarding gender identity discovery, challenges in the transsexualizing process, and difficulties in coming out to their family and society were revealed. This moment helped to understand the uniqueness of each person and their journey of gender reassignment. For many participants, body dysphoria emerged during adolescence, as their minds seemed at odds with their physical bodies and the gender assigned at birth. This feeling was described as a source of distress and isolation until they sought strength in their identified gender.

Participants reported that, despite being aware of the difficulties, they moved forward in the process of seeking to understand their own bodies. Their statements revealed important elements about such experiences and challenges.

*[...] I had already discovered that I was a trans woman, but my restlessness was increasing to the point where I needed to seek help, information, somewhere where I could get more information about the service, [...] (Trans woman, 18 to 29 years old). I knew I needed to go to the health center and that was where everything started, and I went. But the doctor immediately thought it was strange: — What do you mean, you want a referral “for this”? (Trans woman, 18 to 29 years old).*

There was consensus that seeking support services for gender reassignment is lonely and challenging, marked by the lack of knowledge and inability of health professionals to address gender issues. In inland cities and places without specialized services for LGBTQIAPN+, misinformation intensifies the stigma. The participants’ experience revealed that, in many primary healthcare centers, trans people face discrimination due to the professionals’ lack of knowledge, leading them to rely on the internet and social networks for support:

*So, I searched the internet, I didn’t find enough information, until I found an article about the transgender population (Trans woman, 18 to 29 years old). [...] I saw several older trans men recommending looking into the city’s outpatient clinic (Trans man, 18 to 29 years old).*

This stage highlighted that the internet and conversations with friends were the main sources for information about places that provide care for trans people. Some participants indicated that they had to travel long distances to access these places, even crossing state borders to find simpler processes, such as name changes, psychological consultations and prescription refills.

*In healthcare some institutions knew that you had to go to the capital to be able to start the process, but they didn’t tell you (Trans woman, 18 to 29 years old).*

Participants from the Southeast, Northeast and Midwest regions reported that services were better organized with shorter waiting lists. In the North region, the lack of referral services was highlighted, combined with the overload of consultations, which made the process discouraging.

### Category 2: awareness of transgender outpatient clinic services

This category encompassed reports on outpatient services, analyzing access to these spaces, including perceptions about reception, care and challenges faced during appointments. The need for more specialized and qualified professionals to provide care was noted, both in listening to and understanding individuals, in a prejudice-free manner. The need to improve knowledge of the specificities of gender identity of trans men and women was indicated, as well as to expand awareness of the importance of welcoming and respectful care.

*There in the city, it’s an issue with the service because it takes too long […], so I can’t keep my hormone therapy on schedule if they’re delayed by more than a month.” (Trans man, 18 to 29 years old). I think that the communication of the health professionals who provide care is still very precarious in relation to the way in which terms should be used (Trans woman, 39 to 49 years old).*

### Category 3: Communication in transgender outpatient clinics

The preparedness of outpatient clinic professionals to care for the trans population and the availability of informational materials on access to services were explored. The need for specific language, accessible formats of materials and relevant messages to share information about services was discussed. There were reports of a lack of understanding and insecurity among some professionals when dealing with gender identity and sexual orientation issues.

*[...] health workers have no idea of what we are and how we are (Trans man, 30 to 39 years old).*

**Figure 2:**
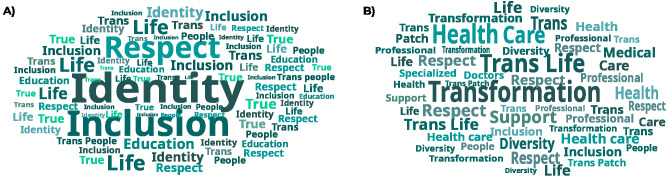
Word cloud from focus group 1 – transgender people (A) and focus group 2 – healthcare professionals (B)

Participants highlighted the scarcity of informational materials in health services. It was revealed that access to these materials occurred mainly through non-governmental organizations. Some communication strategies were suggested, such as the dissemination of digital and audiovisual content, in addition to printed materials and the use of public spaces to distribute information. For these strategies, participants suggested the use of keywords such as “respect” and “resilience”, accompanied by reflections on gender identity and social name. Based on these suggestions, the most relevant terms were organized into a word cloud (Figure 2).

## FOCUS GROUP 2 – HEALTH PROFESSIONALS

### Category 1: “portrait”

After brief introductions, healthcare professionals shared experiences and challenges identified in their respective regions, addressing difficulties in political management and the diversity of local realities. They expressed the desire to improve access to health care for the trans population, despite the structural and management limitations of the SUS in different territories. Despite regional differences, the reports highlighted similarities in the services, such as the high demand for care. The qualification of these services was identified as a challenge because it requires more professionals, better regulation and the formation of integrated networks to facilitate access and meet the specific needs of the trans population.

### Category 2: awareness of transgender outpatient clinic services

This category summarized how people reach transgender outpatient clinics. The main challenges and uncertainties regarding access to healthcare services offered by SUS were discussed from the perspective of professionals. The importance of health professionals understanding the specificities of the trans population and using appropriate languages was reported, as well as the need for further professional development:

*[...] just addressing someone by their name makes a difference, and they are thrilled. (PS3, 30 to 39 years old). [...] from colleagues in care to the reception team, I see a lot of difficulty in understanding sexuality and identity (PS2, 50 years or older).*

### Category 3: Communication in trans outpatient clinics

This category revealed how communication occurs in transgender outpatient clinics, aiming to diagnose the communication of these services. The topics covered included investigating whether there are communication channels for these services aimed at users, how these services are presented, whether a visual identity is adopted to connect with users in the outpatient clinic spaces, and whether there is an internal communication strategy for team interaction.

*About the space, there is nothing that has a trans identity, there is nothing that stands out (PS1, 50 years or older). [...] internal communication here in the municipality is directed and coordinated by health centers, there are six regions, in each region there is an LGBT liaison (PS2, 50 years or older).*

The existence of partnerships to support the visibility of services and improve communication with users was investigated. The most relevant information to be disseminated and the types of materials best suited for transmitting this information to users were identified:

*I believe that social media is in fact very accessible, right? It’s something organic for communication (PS3, 30 to 39 years old).*

Professionals highlighted the need to improve internal communication and training on issues of transsexuality, gender, and LGBTQIAPN+ terminology. Although some services use social media and WhatsApp for interaction, the lack of specialized communication staff makes it difficult to manage and regularly share content. In the outpatient facilities, although there is no planned visual identity, there is signage on the topic and information on the right to a social name. Investment in communication increases during LGBTQIAPN+ thematic dates, but it is insufficient to address specific health issues for trans men and women. The group suggested using terms such as “welcoming,” “respect,” and “professional care” for communication strategy (Figure 2) . Proposed messages included reflections on transformation, self-discovery, and the appreciation of professionals and technicians.

## DEVELOPMENT OF THE COMMUNICATION PLAN

Based on the focus groups, a communication plan was proposed to enhance the visibility of transgender outpatient clinics within the SUS, integrating the needs of trans people and outpatient clinic workers. In this plan, the concept “TRANS Path, Respect, Dignity and True Identity” was collectively developed. Messages for the trans population emphasized the recognition of identity and access to quality services within the SUS. Messages for professionals emphasized the positive impact of services on the lives of trans people and the importance of family support .

**Figure 3:**
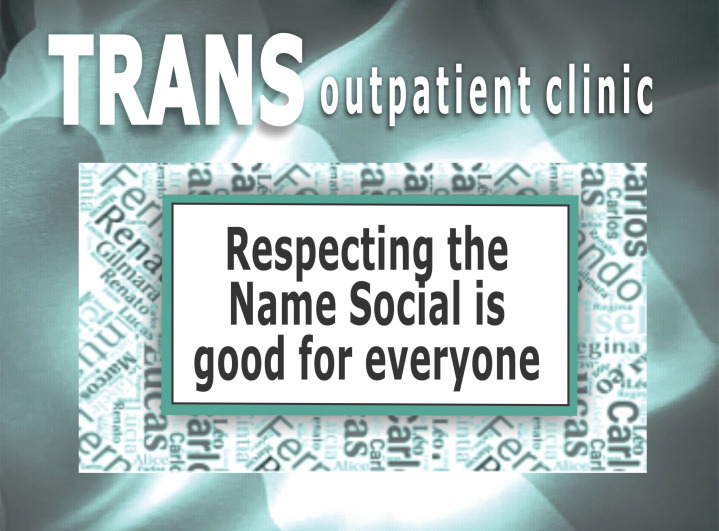
General label: public transgender outpatient clinics

**Figure 4:**
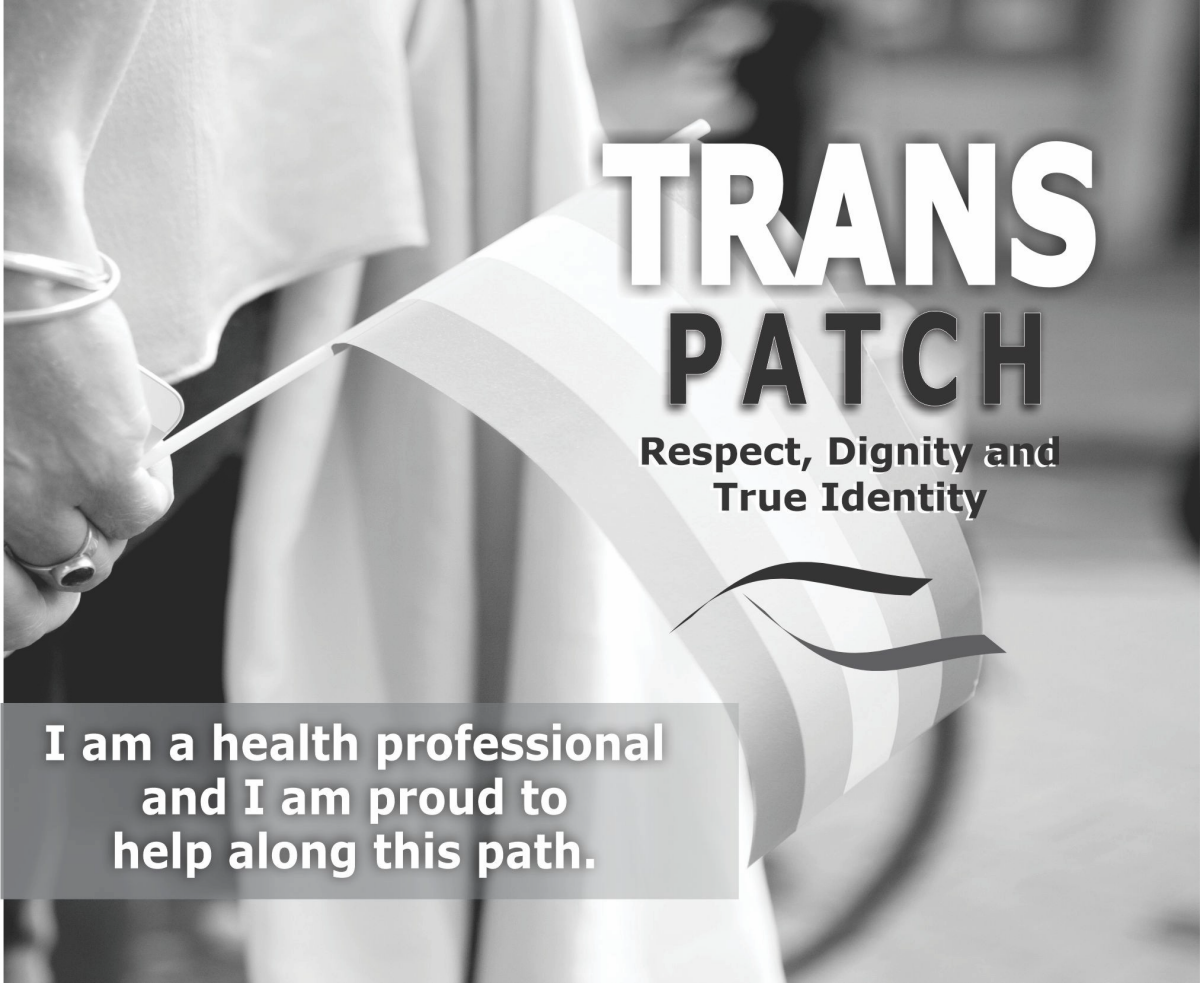
Graphic: support from health professionals for trans people

**Figure 5:**
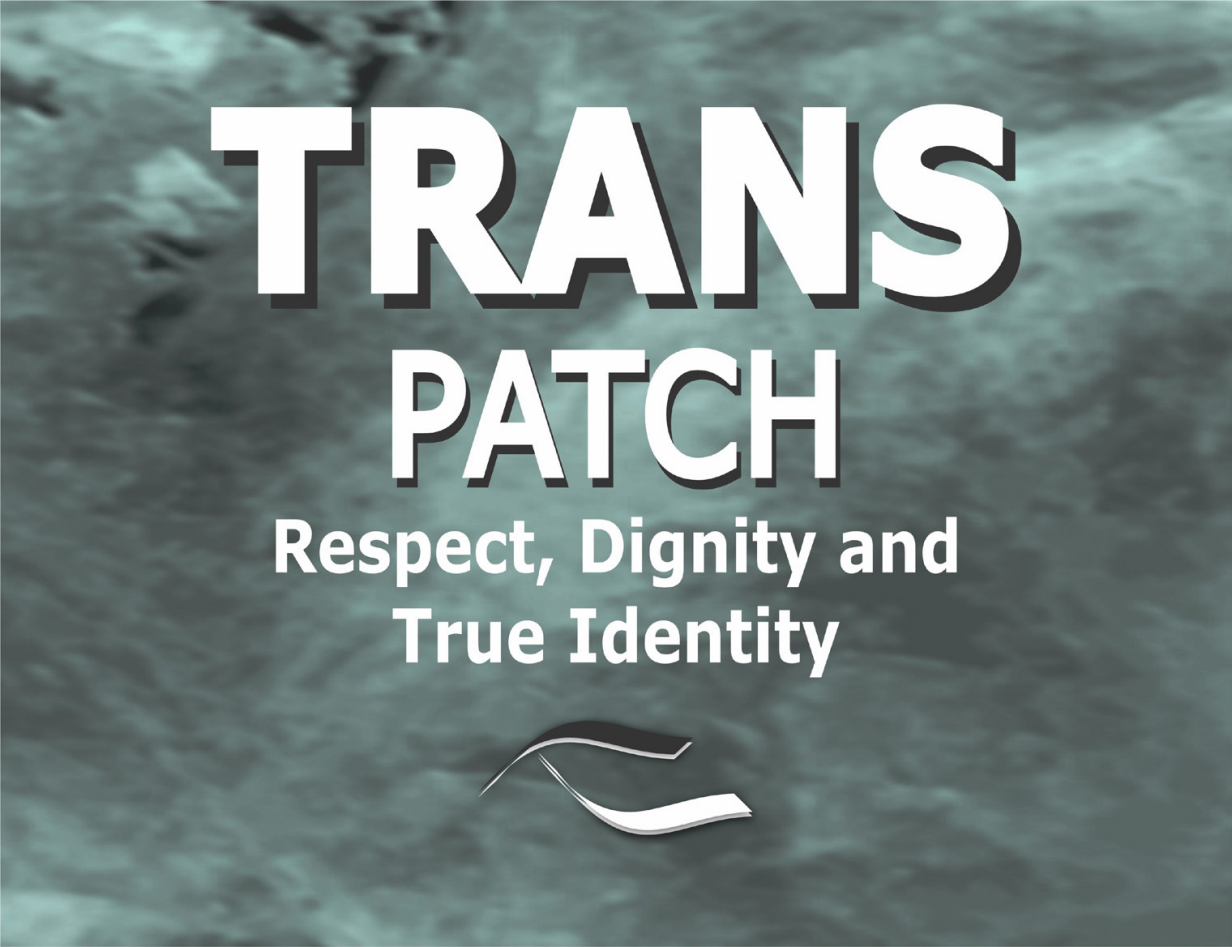
Graphic: Respect, Dignity and True Identity

The proposed plan was discussed in the validation focus group, which unanimously approved the strategy and materials produced. For the mobilization campaign, graphic materials for social media, distribution, and signage were created. These materials (posters, stickers, and flyers) were made available to all participants for printing and dissemination (Figures 3, 4, and 5). The launch took place on National Trans Day of Visibility, with a nationwide social media campaign shared by volunteers across Brazil.

## DISCUSSION

This study aimed to develop dialogic practices between transgender people and healthcare professionals in Brazil, providing a space for sharing experiences and challenges in the transsexualizing process. The study identified significant barriers in communication between users and health services, ranging from lack of awareness of outpatient clinics as free and specialized services to access obstacles exacerbated by stigma and discrimination.

For example, that transvestites and transgender women resort to biomedical technologies to suppress masculine physical traits, seeking to modify their bodies in order to avoid prejudice. This search was associated with risks such as self-medication, self-harm and, in more severe cases, even death.^10^

There was evidence that families of people undergoing gender transition faced difficulties in obtaining adequate information about the transsexualizing process. Both family members and trans people pointed out the lack of healthcare centers that offered adequate support. This forced them to deal with this process independently, without the necessary institutional support.^10^

It is crucial to continuously reflect on transgender health, underscoring that care should not be limited to hormone therapy prescriptions, as it should consider individual and family needs, reproductive desires and issues related to sex work.^11^ It is emphasized that many trans people seek healthcare services in situations of extreme vulnerability. This demands care that goes beyond the desired body changes, which depend on a network of recognition and social inclusion.^12^

The described circumstances highlight the centrality of the relationship between healthcare professionals and users in building mental health care, which requires active listening and the development of a therapeutic plan based on shared responsibility, respecting the autonomy of the people involved.^13^ As evidenced in this study, such a dialogical relationship between professionals and users is essential to ensure effective communication. This must occur from the initial reception in healthcare services and extend throughout the entire transsexualizing process, with the provision of communication materials that respectfully represent the health and well-being needs of the transgender population.

There is a need for more robust public policies to ensure the inclusion of transgender people in the healthcare system,^14^ since this population remains distanced from services due to the lack of specific mechanisms that facilitate access. Interviews conducted with users of transgender outpatient clinics highlighted the urgency of more effective communication and strengthening of these services, which should also reflect the strengthening of the SUS.

Based on the premise that communication can serve as a tool for maintaining or transforming reality,^15^ a communication strategy was developed, widely disseminated and replicated on social media on the National Trans Day of Visibility in Brazil. This aimed to give visibility and a voice to the protagonists of this struggle: transgender people receiving care at transgender outpatient clinics and healthcare professionals. It is expected that these materials will continue to circulate and inspire new communication initiatives, developed collaboratively, together with them (rather than “for them”). This study can contribute to the design of future projects, aimed to improve services for the trans population, especially with regard to health communication.

One limitation of this study was the virtual nature of the focus groups, driven by the COVID-19 socio-health context, which restricted participation due to technological barriers and scheduling constraints, especially for healthcare professionals. The limited timeframe for the research also reduced participation, impacting the representativeness of the data. This study demonstrated that dialogic communication can enhance the visibility of healthcare services, integrating users and professionals in collaborative practices. For instance, it proposes developing communication strategies that guide the transgender population, respecting gender identity and promoting more equitable access to healthcare services.
