# Supplementary material for: Transgender outpatient clinics within the SUS: how can communication enhance service visibility?
Source: Epidemiol Serv Saude. 2024 Jan 10;33(spe1):e2024270. doi: 10.1590/S2237-96222024v33e2024270.especial.en (PMC11734656; doi:10.1590/S2237-96222024v33e2024270.especial.en)
Supplement: Translation

# Translation: Ambulatórios trans do SUS: como a comunicação pode ampliar a visibilização do serviço?

**DOI:** 10.1590/S2237-96222024v33e2024270.especial.pt

**Authors:** Juliana Michelotti Fleck, Salete Saionara Santos Barbosa, Aline Guio Cavaca

**Affiliations:** 1 Ministério da Saúde, Secretaria de Atenção Primária à Saúde, Departamento de Estratégias e Políticas de Saúde Comunitária, Brasília, DF, Brasil; 2 Ministério da Saúde, Secretaria de Vigilância em Saúde e Ambiente, Departamento de HIV/aids, tuberculose e hepatites virais e infecções sexualmente transmissíveis. Brasília, DF, Brasil; 3 Fundação Oswaldo Cruz, Escola de Governo Fiocruz-Brasília. Brasília, DF, Brasil

**Keywords:** Comunicação em Saúde, Transexualidade, Vulnerabilidade em Saúde, Sistema Único de Saúde, Pessoas Transgênero

## Abstract

**Objetivo:**

Mapear dificuldades de comunicação nos serviços de saúde para pessoas transexuais, propondo estratégias para aumentar a visibilidade desses ambulatórios e melhorar o acesso a estes.

**Métodos:**

Trata-se de estudo quanti e qualitativo. Foram aplicados 44 questionários *online* e realizados dois grupos focais com pessoas trans e com profissionais de saúde em 2021.

**Resultados:**

O desconhecimento sobre a gratuidade e especialidade dos serviços e o estigma e a discriminação sofridos pela população trans foram as principais dificuldades identificadas com relação ao acesso aos serviços de saúde. Com base nesse diagnóstico, foi elaborado um plano de comunicação adaptado às necessidades de cada público. A estratégia foi divulgada no Dia Nacional da Visibilidade Trans e enfatizou a mensagem “Caminho TRANS. Respeito, Dignidade e Identidade de Verdade”.

**Conclusão:**

O desenvolvimento de estratégias de comunicação de maneira dialógica pode aumentar a visibilidade dos serviços dos ambulatórios trans. Faz parte dessa estratégia a integração de usuários e profissionais em práticas colaborativas, respeitando a identidade de gênero e promovendo o acesso mais equitativo aos serviços.

## Contribuições do estudo

**Principais resultados:**

Desenvolveu-se de forma dialógica, junto aos profissionais e aos usuários dos serviços de saúde nos ambulatórios trans, estratégia comunicacional divulgada nas redes sociais no Dia Nacional da Visibilidade Trans. Isso deu visibilidade e voz a esses indivíduos.

**Implicações para os serviços:**

Os serviços de saúde devem ampliar a divulgação sobre a gratuidade e especialização dos ambulatórios trans, capacitar profissionais contra o estigma, facilitar o acesso dos usuários e adotar a comunicação dialógica que respeite a identidade de gênero.

**Perspectivas:**

A expansão de iniciativas colaborativas com a população trans capazes de promover políticas inclusivas pode contribuir para a visibilidade e o acesso equitativo aos serviços de saúde.

## INTRODUÇÃO

O termo “população trans” abrange pessoas cuja identidade de gênero difere do sexo de nascença. Trata-se de experiência interna e individual do gênero de cada pessoa. Essa experiência inclui a percepção pessoal do corpo que pode ser acompanhada de modificações físicas por meios médicos, cirúrgicos ou outros, além de expressões de gênero como vestimentas e maneirismos.^1^

As pessoas transexuais e transgênero fazem parte de grupo diverso e vulnerável. Elas enfrentam sérios desafios, o que inclui a expectativa de vida média de 35 anos. Contribuem para esse cenário preocupante problemas de saúde mental, maior risco de contaminação com HIV e outras infecções sexualmente transmissíveis e estressores sociais, como violências e dificuldades de emprego e acesso à saúde, contexto que eleva a probabilidade de comportamento suicida.^2^

Devido ao desconhecimento dos profissionais sobre transexualidade, as experiências negativas de pessoas trans nos serviços de saúde são frequentes,^3^ potencializadas pela comunicação difícil e pelas barreiras de aceitação. É evidente a relevância da oferta de atendimentos especializados que compreendam a necessidade de transformação corporal e o percurso transexualizador como processos de saúde e doença. Soma-se a isso a importância da qualificação de profissionais para atender às necessidades desses indivíduos nos serviços públicos de saúde.^4^

Em 2013, criou-se a linha de cuidado de atenção aos usuários e às usuárias em processo transexualizador no Sistema Único de Saúde (SUS).^5^ Tal estrutura da atenção à saúde inclui a atenção básica e a atenção especializada. A atenção básica coordena o cuidado contínuo, e a atenção especializada é responsável por pontos de serviços urgentes, ambulatoriais e hospitalares. Isso envolve acompanhamento clínico e pré e pós-operatório, hormonioterapia e cirurgias. Os ambulatórios trans do SUS oferecem serviços essenciais e espaços de apoio para a população em processo de transição ou em busca de informações para aceitação própria. Há a oferta de suporte de psicólogos, fonoaudiólogos, nutricionistas, endocrinologistas, psiquiatras e assistentes sociais, incluindo acompanhamento para terapia hormonal.^5^

Existem desafios para alcançar a excelência, apesar da importância desses serviços. Destacam-se as dificuldades comunicacionais entre profissionais de saúde e usuários nos ambulatórios trans, a exemplo das diferenças linguísticas, das limitações físicas, da imposição de valores e das influências inconscientes.^6^ Diferenças socioculturais, estigmas e preconceitos complexificam o processo. Este estudo objetivou identificar essas dificuldades e propor estratégias de comunicação para promover a visibilidade dos ambulatórios trans e facilitar o acesso aos serviços oferecidos.

## MÉTODOS

Trata-se de pesquisa quanti e qualitativa de caráter exploratório. Esta foi executada em cinco etapas: envio de convites direcionados para anuência da participação e aplicação de questionário, realização de grupos focais, relatoria dos pontos mais significativos e análise qualitativa dos grupos realizados, planejamento e propostas de material de comunicação e validação dos materiais produzidos (Figura 1).

**Figura 1:** Síntese metodológica da pesquisa.

### Local e população de estudo

O estudo envolveu dois grupos: pessoas trans que buscavam atendimento de saúde no SUS e profissionais de saúde que trabalhavam em ambulatórios trans e centros de referência. Os participantes foram selecionados com base em informações coletadas nos portais do Ministério da Saúde, de ambulatórios trans, hospitais e organizações não governamentais LGBTQIAP+. Os contatos iniciais foram direcionados a indivíduos das cinco regiões do país. Sujeitos potenciais foram convidados a partir da indicação dos participantes iniciais. Isso ocorreu com base na técnica de amostragem “bola de neve”.^7^

Os critérios de inclusão para pessoas trans foram: ser homem ou mulher trans, usuário ou não dos serviços de saúde, selecionado por indicação dos ambulatórios ou de redes organizadas de pessoas trans. O critério de inclusão para os profissionais de saúde foi ter vínculo empregatício com o ambulatório trans do SUS, como médico, enfermeiro, assistente de enfermagem, psicólogo ou assistente social. Pessoas trans que ainda não haviam completado 18 anos de idade e profissionais de saúde que atuassem apenas como voluntários nos serviços dos ambulatórios trans foram excluídos do estudo. 

Em virtude do contexto sociossanitário da pandemia de covid-19, os contatos com os participantes e os encontros foram realizados inicialmente por *e-mail*, confirmados pelo aplicativo de conversa WhatsApp e por contato telefônico. Os grupos focais aconteceram *online* na plataforma Zoom. Após o convite e o aceite confirmado, os participantes foram orientados a preencher questionário *online*, enviado 15 dias antes da realização dos grupos focais, e assinar o Termo de Consentimento Livre e Esclarecido (TCLE).

### Coleta de dados

Encaminhou-se questionário via Google Forms por *e-mail* e WhatsApp para obter informações sobre o perfil dos interessados e suas perspectivas sobre o tema da pesquisa. 

Foram recebidas 9 respostas de profissionais de saúde e 19 de pessoas trans após o envio de 89 e 25 convites. Os questionários, com questões abertas e de múltipla escolha, foram adaptados para cada grupo. Eles abordavam aspectos como identificação, localização, idade, educação, área de atuação, utilização e atuação nos serviços dos ambulatórios e tipo de comunicação utilizada nos serviços do ambulatório trans. 

Todos os respondentes convidados foram distribuídos em dois grupos focais: um com pessoas trans (grupo focal 1) e outro com profissionais de saúde (grupo focal 2). O grupo focal 1 contou com a presença de 12 pessoas, de quatro regiões do país (Centro-Oeste, Sudeste, Norte e Nordeste), além de duas moderadoras e um suporte técnico. O grupo focal 2 contou com a participação de três profissionais de saúde do Centro-Oeste, Sudeste e Nordeste, além de duas moderadoras e uma profissional para apoio e suporte técnico. Os grupos focais realizaram-se virtualmente, com duração aproximada de duas horas e trinta minutos. O estudo seguiu as orientações do Ofício Circular n. 2/2021 da Comissão Nacional de Ética em Pesquisa^9^ para pesquisa em ambientes virtuais. A pesquisa foi aprovada pelo Comitê de Ética em pesquisa da Fiocruz Brasília (CAAES 50379321.1.0000.8027)**,** garantindo ética, confidencialidade e esclarecimento sobre os procedimentos.

### Análise dos dados

Analisaram-se os dados dos questionários descritivamente para caracterizar a população estudada e submeteram-se as transcrições dos grupos focais à análise temática de conteúdo para interpretar as comunicações.^8^ Foram comparadas as impressões e experiências dos participantes sobre histórias de vida, ambientes dos ambulatórios e comunicação. Os temas comuns incluíram história do participante, conhecimento dos serviços dos ambulatórios trans e comunicação. 

### Desenvolvimento do plano de comunicação 

Os grupos focais identificaram pontos-chave na comunicação, lacunas de informação e estratégias eficazes para aumentar a visibilidade dos ambulatórios trans. Com base nesses resultados, elaborou-se o plano de comunicação com materiais e estratégias de engajamento para instituições, usuários e profissionais. Foi sugerida uma mobilização nacional pelas redes sociais, liderada pelos participantes dos grupos focais, cujas mensagens foram compartilhadas no perfil @comunicatrans no Instagram.

### Validação dos materiais produzidos

A validação do plano de comunicação ocorreu em encontro virtual durante duas horas, via Microsoft Teams, com um grupo de 12 pessoas que incluiu usuários e profissionais de saúde dos grupos focais. A estratégia de comunicação e o conceito da campanha foram apresentados, e houve sugestões para mobilização e peças para compartilhamento. Divulgaram-se as peças, com avaliação e ajustes propostos pelos participantes, e estas foram acompanhadas de debate sobre a aplicabilidade dos materiais.

## RESULTADOS 

### Breve descrição dos participantes

Segundo a análise dos 19 questionários da população trans, 5 respondentes eram homens trans, e 14, mulheres trans, sendo a maioria jovem (11 indivíduos entre 18 e 39 anos). Todos já eram usuários do SUS, sendo 14 usuários dos ambulatórios trans. Três respondentes não tinham conhecimento dos serviços nesses ambulatórios. A escolaridade média desse grupo era ensino superior completo. Duas pessoas tinham concluído somente o ensino médio. 

Conforme os questionários dos 9 profissionais de saúde, todos estavam vinculados diretamente aos ambulatórios trans. Cinco eram mulheres cisgênero, e 4, homens cisgênero. Quatro respondentes tinham entre 30 e 39 anos, e 4 tinham 50 anos ou mais. Dois respondentes eram médicos, e os demais relataram atuação em áreas afins. 

## GRUPO FOCAL 1 – PESSOAS TRANS

### Categoria 1: “retrato”

A atividade começou com a categoria “retrato”. Nesta, cada participante se apresentou e compartilhou suas histórias de vida. Foram reveladas experiências pessoais sobre a descoberta da identidade de gênero, desafios no processo transexualizador e dificuldades de se assumir para a família e a sociedade. Esse momento contribuiu para a compreensão das singularidades de cada pessoa e da sua jornada de readequação de gênero. Para muitos participantes, já na adolescência, surgiu a dificuldade de aceitação do próprio corpo, e a mente não parecia estar de acordo com o corpo físico e o sexo declarado no nascimento. Tal sentimento foi retratado como fator de angústia e recolhimento, até decidirem buscar fortalecimento na identidade com que se identificavam.

Os participantes relataram que, mesmo cientes das dificuldades, avançaram no processo em busca da compreensão do próprio corpo. Os depoimentos revelaram elementos importantes sobre tais vivências e desafios.

*[...] eu* *já tinha me descoberto mulher trans, só que a minha inquietude estava aumentando a ponto de procurar ajuda, informação, algum veículo onde pudesse ter mais informações sobre o serviço, [...]* (Mulher trans, 18 a 29 anos). *Eu soube que precisava ir no postinho de saúde e que lá era o início de tudo, e fui. Só que a médica na mesma hora estranhou: — Como assim, você quer encaminhamento “para isso”?* (Mulher trans, 18 a 29 anos).

Houve consenso de que a busca por serviços de apoio para a readequação de gênero é solitária e desafiadora, marcada pela falta de conhecimento e inabilidade dos profissionais de saúde diante de questões de gênero. Em cidades do interior e locais sem serviços especializados para LGBTQIAPN+, a desinformação intensifica o estigma. A experiência dos participantes revelou que, em muitas unidades básicas de saúde, pessoas trans enfrentam discriminação decorrente do desconhecimento dos profissionais e passam a recorrer à internet e às redes sociais em busca de suporte.

*Então eu procurei na internet, não encontrei informações suficientes, até que encontrei um artigo, falando sobre a população transgênero* (Mulher trans, 18 a 29 anos). *[...] eu vi bastante homens trans, mais velhos, indicando pesquisar sobre o ambulatório da cidade* (Homem trans, 18 a 29 anos).

Essa etapa explicitou que a internet e as conversas com amigos foram as principais fontes na busca por informações sobre locais de atendimento às pessoas trans. Alguns participantes indicaram que foi necessário percorrer longos deslocamentos para chegar a esses locais, cruzando até limites estaduais por acessos simplificados, como orientação para mudança de nome, consultas psicológicas e dispensação de receituários.

*Na saúde, alguns órgãos até sabiam que tinha que ir para a capital para poder iniciar o processo,* só que não falavam (Mulher trans, 18 a 29 anos).

Participantes das regiões Sudeste, Nordeste e Centro-Oeste relataram que os serviços estavam mais organizados, e as filas de espera eram menores. No Norte, foi apontada a falta de serviços de referência, somada à sobrecarga nas consultas, o que tornava o processo desestimulante. 

### Categoria 2: conhecimento dos serviços dos ambulatórios trans

Esta categoria agregou relatos a respeito dos serviços dos ambulatórios, tendo sido analisado o acesso a esses espaços, incluindo percepções sobre recepção, acolhimento e dificuldades no atendimento. Registrou-se a necessidade de mais profissionais especializados e qualificados para o atendimento, tanto na escuta como na compreensão dos indivíduos, de forma livre de preconceitos. Indicou-se a necessidade de melhorar o conhecimento das especificidades de identidade de gênero de homens e mulheres trans, bem como ampliar o entendimento da importância do atendimento acolhedor e respeitoso.

*Lá na cidade* é *uma questão do atendimento, porque demora [...], então eu não consigo manter a hormonização em dia se eles atrasam um mês e pouco* (Homem trans, 18 a 29 anos). *Eu acho que a comunicação dos profissionais de saúde que nos atende ainda é muito precária em relação à maneira dos termos a serem usados* (Mulher trans, 39 a 49 anos).

### Categoria 3: comunicação nos ambulatórios trans

Exploraram-se o preparo dos profissionais dos ambulatórios para atender a população trans e a disponibilidade de materiais informativos sobre acesso aos serviços. A necessidade de linguagem específica, formatos acessíveis de materiais e mensagens relevantes para compartilhar informações sobre os serviços foi discutida. Houve relatos sobre a falta de compreensão e a insegurança de alguns profissionais diante de questões de identidade de gênero e orientação sexual.

*[...] todos os trabalhadores de saúde não têm o menor entendimento do que nós somos e como somos* (Homem trans, 30 a 39 anos).

Os participantes destacaram a escassez de materiais informativos nos serviços de saúde. Revelou-se que o acesso a esses materiais ocorreu principalmente por meio de organizações não governamentais. Algumas estratégias de comunicação foram sugeridas, a exemplo da divulgação de conteúdos digitais e audiovisuais, além de materiais impressos e do uso de espaços públicos para distribuição de informações. Para essas estratégias, os participantes indicaram a utilização de palavras-chave como “respeito” e “resistência”, acompanhadas de reflexões sobre identidade de gênero e nome social. Com base nessas sugestões, os termos mais relevantes foram organizados em nuvem de palavras (Figura 2).

**Figura 2:** Nuvem de palavras do grupo focal 1 – pessoas trans (A) e grupo focal 2 – profissionais de saúde (B)

## GRUPO FOCAL 2 – PROFISSIONAIS DE SAÚDE 

### Categoria 1: “retrato”

Após breve apresentação, os profissionais compartilharam experiências e desafios identificados em suas respectivas regiões, abordando dificuldades na gestão política e a diversidade de realidades locais. Eles expressaram o desejo de melhorar o acesso à saúde para a população trans, apesar das limitações estruturais e de gestão do SUS em diferentes territórios. A despeito das diferenças regionais, os relatos destacaram semelhanças nos serviços, como a grande demanda por atendimento. A qualificação desses serviços foi apontada como um desafio por exigir mais profissionais, melhor regulação e formação de redes integradas para facilitar o acesso e atender às necessidades específicas da população trans.

### Categoria 2: conhecimento dos serviços dos ambulatórios trans

Esta categoria sintetizou como as pessoas chegam aos ambulatórios trans. Foram expostas as principais dificuldades e dúvidas sobre o acesso aos serviços de saúde ofertados pelo SUS sob a perspectiva do profissional. Relatou-se a importância de os profissionais de saúde compreenderem as especificidades da população trans e as linguagens apropriadas e de buscarem qualificação para a atenção e o atendimento a esse público.

*[...] o simples fato de você acolher a pessoa pelo seu nome é diferente e a pessoa fica maravilhada* (PS3, 30 a 39 anos). *[...] desde colegas da assistência, até a equipe da recepção, eu vejo muita dificuldade em compreender de sexualidade, identidade* (PS2, 50 anos ou mais).

### Categoria 3: comunicação nos ambulatórios trans

Esta categoria revelou como se dá a comunicação nos ambulatórios trans, visando ao diagnóstico sobre a comunicação desses serviços. Os temas abordados incluem investigar se existem canais de comunicação desses serviços voltados aos usuários, como são apresentados esses serviços, se é adotada identidade visual que crie laços com os usuários nos espaços do ambulatório e se há estratégia de comunicação interna para interlocução entre as equipes.

*Sobre o espaço, não tem nada que tenha uma identidade trans, não tem nada que destaque* (PS1, 50 anos ou mais). *[...] a comunicação interna aqui no município é dirigida e coordenada por unidades de saúde, são seis regiões, em cada região há uma interlocutora LGBT* (PS2, 50 anos ou mais).

Investigou-se a existência de parcerias para apoiar a visibilidade dos serviços e melhorar a interlocução com a população usuária. Foram levantadas as informações mais relevantes a serem disseminadas e os tipos de materiais mais adequados e eficientes para a transmissão junto aos usuários.

*Eu acredito que as redes sociais, é de fato muito acessível, né. É algo muito orgânico para a comunicação* (PS3, 30 a 39 anos).

Os profissionais destacaram a necessidade de aprimorar a comunicação interna e a qualificação em questões de transexualidade, gênero e terminologias LGBTQIAPN+. Embora alguns serviços utilizem redes sociais e WhatsApp para interação, a falta de funcionários especializados em comunicação dificulta a administração e o compartilhamento regular de conteúdo. Nas instalações dos ambulatórios, apesar de não haver a prevalência de uma identidade visual planejada, há sinalização sobre o tema e informações sobre o direito ao nome social. O investimento em comunicação aumenta durante datas temáticas LGBTQIAPN+, mas é insuficiente para abordar questões de saúde específicas para homens e mulheres trans. O grupo destacou “acolhimento”, “respeito” e “atendimento profissional” como palavras sugeridas para a estratégia de comunicação (Figura 2). As mensagens de conexão propostas incluíram reflexões sobre transformação, encontro consigo mesmo e valorização dos profissionais e técnicos. 

### Desenvolvimento do plano de comunicação

Com base nos grupos focais, foi proposto plano de comunicação para qualificar a visibilidade dos ambulatórios trans do SUS, integrando necessidades das pessoas trans e dos trabalhadores dos ambulatórios. Nesse plano, desenvolveu-se coletivamente o conceito “Caminho TRANS. Respeito, Dignidade e Identidade de Verdade”. As mensagens para a população trans destacaram o reconhecimento da identidade e o acesso aos serviços de qualidade no SUS. As mensagens para profissionais enfatizaram o impacto positivo dos serviços na vida das pessoas trans e a importância do apoio familiar.

Discutiu-se o plano proposto no grupo focal de validação, que aprovou unanimemente a estratégia e os materiais produzidos. Para o desenvolvimento da ação de mobilização, produziram-se materiais gráficos para redes sociais, distribuição e sinalização. Os materiais (cartazes, adesivos e fôlderes) foram disponibilizados a todos os participantes para impressão e divulgação (Figuras 3, 4 e 5). O lançamento ocorreu no Dia Nacional da Visibilidade Trans, com mobilização nacional nas redes sociais, tendo sido compartilhado por voluntários em todo o Brasil.

**Figura 3:** Marca geral: público ambulatórios trans

**Figura 4:** Peça: apoio dos profissionais de saúde às pessoas trans

**Figura 5:** Peça: Respeito, Dignidade e Identidade de Verdade

## DISCUSSÃO

Esta pesquisa objetivou desenvolver a prática dialógica entre pessoas trans e profissionais dos serviços de saúde do Brasil, proporcionando espaço para compartilhar experiências e desafios no processo de transexualização. O estudo identificou barreiras significativas na comunicação entre usuários e serviços de saúde, desde o desconhecimento dos ambulatórios como serviço gratuito e especializado até os obstáculos de acesso, potencializados pelo estigma e pela discriminação.

Soube-se, por exemplo, que travestis e mulheres transexuais recorrem a tecnologias biomédicas para suprimir traços corporais considerados masculinos, buscando modificar características em seus corpos para evitar o preconceito. Essa busca associou-se a riscos como automedicação, automutilação e, em casos mais graves, até mesmo à morte.^10^

Houve evidências de que famílias de pessoas em transição de gênero enfrentavam dificuldades para obter informações adequadas sobre o processo de transexualização. Tanto os familiares quanto as pessoas trans apontaram a carência de unidades de saúde que ofereceram suporte adequado. Isso as obrigou a lidar com tal processo de forma independente, sem o devido apoio institucional necessário.^10^

Ressalta-se a importância de reflexão contínua sobre a saúde das pessoas trans e reitera-se que o cuidado não deve se limitar à prescrição de hormonização, uma vez que deve considerar demandas individuais e familiares, desejos reprodutivos e questões relacionadas ao trabalho sexual.^11^ Sublinha-se que muitas pessoas trans chegam aos serviços de saúde em situações de extrema vulnerabilidade. Isso demanda cuidados que ultrapassam as modificações corporais desejadas, os quais dependem de rede de reconhecimento e inclusão social.^12^

As circunstâncias descritas revelaram ser central a relação entre profissionais e usuários na construção do cuidado em saúde mental, que requer escuta ativa e elaboração de projeto terapêutico baseado na responsabilidade compartilhada, em respeito à autonomia das pessoas envolvidas.^13^ Conforme evidenciado neste estudo, tal relação dialógica entre profissionais e usuários é essencial para garantir a comunicação qualificada. Isso deve ocorrer desde o processo de acolhimento nos serviços de saúde até a totalidade do processo transexualizador, com disponibilização de materiais de comunicação que representem dignamente a população trans em suas necessidades de saúde e bem-estar.

Existe a necessidade de políticas públicas mais robustas para garantir a inclusão de pessoas trans no sistema de saúde,^14^ pois essa população se encontra distanciada dos serviços devido à ausência de mecanismos específicos que facilitem o acesso. As entrevistas realizadas com usuários dos ambulatórios trans destacaram a urgência da comunicação mais eficaz e do fortalecimento desses serviços, a refletir também o fortalecimento do SUS. 

Com base na premissa de que a comunicação pode ser um instrumento de manutenção ou transformação da realidade,^15^ desenvolveu-se a estratégia comunicacional amplamente divulgada e replicada nas redes sociais no Dia Nacional da Visibilidade Trans no Brasil. Isso ocorreu para dar visibilidade e voz aos protagonistas dessa luta: as pessoas trans atendidas nos ambulatórios trans e os profissionais de saúde. Espera-se que esses materiais continuem a circular e inspirem novas iniciativas comunicacionais, desenvolvidas de forma colaborativa, junto com eles e elas (e não “para eles e elas”). Este estudo pode contribuir para a construção de futuros projetos que visem aperfeiçoar serviços voltados para a população trans, principalmente no que tange à comunicação em saúde.

A realização dos grupos focais de maneira virtual, devido ao contexto sociossanitário da covid-19, foi fator limitante deste estudo. Esse contexto restringiu a participação por barreiras tecnológicas e de agenda dos envolvidos, principalmente dos profissionais de saúde. O tempo restrito para a pesquisa reduziu a participação, o que impactou na representatividade dos dados. 

Este estudo evidenciou que a comunicação realizada de maneira dialógica pode aumentar a visibilidade dos serviços, integrando usuários e profissionais em práticas colaborativas. Como exemplo, visa-se ao desenvolvimento de estratégias de comunicação que orientem a população trans, respeitando a identidade de gênero e promovendo o acesso mais equitativo aos serviços de saúde.
